# Surgeons are deeply affected when patients are diagnosed with prosthetic joint infection

**DOI:** 10.1371/journal.pone.0207260

**Published:** 2018-11-28

**Authors:** Charlotte Mallon, Rachael Gooberman-Hill, Ashley Blom, Michael Whitehouse, Andrew Moore

**Affiliations:** 1 Musculoskeletal Research Unit, Department of Translational Health Sciences, Bristol Medical School, University of Bristol, Bristol, United Kingdom; 2 National Institute for Health Research Bristol Biomedical Research Centre, Bristol Medical School, University of Bristol, Bristol, United Kingdom; Indiana University, UNITED STATES

## Abstract

Knee replacement is a common preference sensitive quality-of-life procedure that can reduce pain and improve function for people with advanced knee arthritis. While most patients improve, knee replacement surgery has the potential for serious complications. Prosthetic knee infection is an uncommon but serious complication. This study explored the impact of cases of prosthetic knee infection on surgeons’ personal and professional wellbeing. Qualitative telephone interviews were conducted with consultant orthopaedic surgeons who treated patients for prosthetic knee infection in one of six high-volume NHS orthopaedic departments. Data was audio-recorded, transcribed and analysed thematically. Eleven surgeons took part. Analysis identified three overarching themes: (i) At some point infection is inevitable but surgeons still feel accountable; (ii) A profound emotional impact and (iii) Supporting each other. The occurrence of prosthetic joint infection has a significant emotional impact on surgeons who report a collective sense of devastation and personal ownership, even though prosthetic joint infection cannot be fully controlled for. Surgeons stressed the importance of openly discussing the management of prosthetic joint infection with a supportive multidisciplinary team and this has implications for the ways in which orthopaedic surgeons may be best supported to manage this complication. This article also acknowledges that surgeons are not alone in experiencing personal impact when patients have infection.

## Introduction

Knee replacement surgery is a discretionary, preference sensitive surgery that can improve quality of life for people with advanced knee arthritis. It has the potential to improve patients’ quality of life by reducing pain and improving function, but there is also potential for serious complications. Prosthetic joint infection (PJI) is a potentially life-threatening condition associated with severe pain, functional difficulties and poor quality of life [1–3]. If left untreated, PJI may result in limb amputation or death [4,5]. When PJI is identified, further major operations are often required to clear the infection, which reduces the need for subsequent joint removal or even limb amputation. Surgical treatment can be prolonged sometimes involving multiple surgeries and hospitalisations and prolonged antibiotic treatment. PJI and its treatment has a profoundly negative impact on all aspects of patients' lives, including physical, social, emotional and economic aspects and patients undergoing treatment can experience feelings of isolation and insecurity, and an inability to live independently [1,2]. Cahill and colleagues found that the profoundly negative impact on patients’ social functioning and mental health is notable with this group of patients rating their overall quality of life as poor with 12% of patients rating their current situation as equivalent to or worse than death [3].

Infection rates have been reduced by addressing contributory health system and surgical factors [6]. Recent evidence synthesis has shown that multiple patient related factors are associated with developing post-operative infection [7]. However, despite the small risk of adverse events such as infection, patients still have the capacity to benefit greatly from arthroplasty.

Although recent research has concentrated on the impact of infection on patients [1,2], little is known about how surgeons may be affected when patients whom they have treated have infection. Adverse events may have considerable impact on healthcare professionals, particularly if the professionals are seen to be accountable for the outcome [8]. For surgeons, adverse events and surgical complications are a major source of unpleasant emotions, cognitive dysfunction and negative feelings [9]. A growing body of evidence describes how adverse events and medical errors have a negative emotional impact on surgeons and other healthcare professionals [10–15].

Many surgeons uphold a “surgical ideal”, whereby surgery is seen as the only chance to cure a patient or improve their quality of life. When surgery does not achieve this goal surgeons report feelings of failure which may impact on their coping response to intraoperative complications [9]. Surgeons across all specialties are largely unprepared for the intense emotional responses that adverse events can elicit, which may not only affect clinical judgement, but also impact on surgeons’ personal and professional identity [10]. These emotional responses to adverse events can be associated with increased feelings of personal responsibility, guilt, self-doubt, anxiety and distress [12,13], but are dependent on factors including surgical experience, colleagues’ reactions and the preventability of complications [14].

There is an element of uncertainty in surgery where clear scientific evidence is lacking [15]. While adverse events can impact decision-making by affecting judgement or changing practice [14], the “second victim” [16] of medical mistakes or adverse occurrence–surgeons—can feel unique, vulnerable and isolated in their reactions to such events [9,10]. This “oppressive experience” [9] may help to explain surgeons’ tendency to become more conservative and more risk-averse following adverse events [14]. However, there is little literature that focuses on the impact on surgeons, where there is no obvious cause to adverse events such as prosthetic joint infection (PJI).

Approximately 1% of patients who undergo knee replacement develop PJI [17]. In 2015, just over 6,000 knee revision procedures were performed in the UK, of which nearly one quarter (1,420 (23%)) were for infection [18]. Although PJI has been described as “an orthopaedic surgeon’s worst nightmare” [19], relatively little is known about how PJI impacts surgeons professionally and personally. Identification and acknowledgement of the emotional impact of PJI may help in the development of support strategies and maintaining surgeons’ well-being [20], and a deeper understanding of the personal and professional impact of adverse events is needed [11]. Understanding how surgeons react to the occurrence of PJI is an important clinical question and has wider relevance to establishing how surgical teams can be supported during the management of this complication.

Qualitative methods are well established. They are used widely in health research and are recognized as the most appropriate method of understanding complex social interactions, behaviours and perceptions [21–25]. Previous qualitative research in surgery has focused on adverse events and “medical mishaps” [10,12,14, 26,27] and the emotional experience of being a surgeon [9]. To our knowledge, none has focused on adverse events that have no obvious cause, and the impact on surgeons personal and professional wellbeing.

## Methods

### Setting and sampling

In this study we conducted qualitative telephone interviews with Orthopaedic Surgeons treating knee PJI at one of six high-volume UK NHS orthopaedic hospitals. The interviews focussed on surgeons’ experiences of treating knee PJI. We employed a purposive sampling strategy to ensure that participants had experience of relevance to the study [28]. Surgeons were eligible to take part if they were Consultant Orthopaedic Surgeons treating knee PJI at one of six high-volume UK NHS orthopaedic hospitals. Using established clinical networks, orthopaedic surgeons who regularly conduct revision surgery for prosthetic knee joint infection were identified through professional and clinical networks by members of the study team (also consultant surgeons who specialise in this area) and contacted by a researcher (CM, not previously known to the surgeons) and sent an information pack, including a letter of invitation, information booklet, reply form and pre-paid envelope. Surgeons who returned a reply form expressing an interest in participating were contacted by the researcher to discuss the study and to arrange an interview if they wished to participate. Between April 2016 and December 2016, 15 surgeons were invited to participate. Thirteen surgeons expressed an interest in participating in the study. Eleven surgeons provided their written informed consent to audio-recording of the interview and publication of anonymised quotations. Two surgeons who did not return their consent forms could not be contacted and so could not be interviewed. Data saturation was achieved after 11 participants had been interviewed. This was demonstrated by no new themes emerging from the data [29]. At this point, no further interviews were arranged.

### Data collection and analysis

Telephone interviews were conducted by an experienced qualitative researcher not previously known to the participants (CM). The topic guide included key questions focused on the treatment of knee PJI and outcomes after surgery. Probes and prompts were used flexibly, to ensure that participants had the opportunity to expand on areas they considered important. Interviews lasted from 20 to 45 minutes each (mean 32 minutes). Interviews were transcribed, anonymised and imported into the qualitative data management software QSR NVivo 10 [30]. All participants’ names were replaced with pseudonyms, and identifiable information was removed.

Using thematic analysis [31], the researcher (CM) read and re-read the data to ensure familiarity, coding inductively before sorting coded data into themes [32]. Codes were checked for consistency and validation by a second researcher (AM), and the study team (AM, CM, AB, RGH, MW) met to discuss and agree themes [33].

Ethical approval was granted by NRES Committee South West—Exeter (14/SW/0072).

## Results

Eleven surgeons were interviewed. Experience as an orthopaedic consultant varied from one month to 20 years (mean 9.5 years). Our findings show the personal and professional impact of knee PJI on surgeons, and how, despite their expertise, surgeons remain vulnerable to cases of PJI, perceiving it as inevitable at some point in their career. Analysis of the data suggests that regardless of the variation in surgeons’ years of experience, knee PJI is a major concern, and is devastating for surgeons, as well as their patients. Three themes characterise surgeons’ views and experiences of treating knee PJI, and illustrative quotations are referred to throughout (Tables 1–3).

**Table 1:** 

**Theme 1: At some point infection is inevitable but surgeons still feel accountable.**	I think the first time it happens, it’s very hard to deal with as a surgeon, but actually, we do a job that involves risks and involves complications associated with the surgery, and as long as you are comfortable with yourself that you have taken every measure you can to minimise those risks at the time of their initial surgery, you need to accept that, it’s not your fault. Actually, there are a certain proportion of patients, that despite every measure being taken, does develop an infection and it doesn’t just happen to you; it happens to every surgeon that does that surgery, and all you can do in that situation is be empathic with your patient, do everything that you possibly can, to treat them in the way you would wish to be treated yourself and do what you can to try to clear it. (Surgeon 1) It's, it's not good news. And as I said, it's extremely rare and my experience I have never, ever had an infection in a primary joint as an early infection. And this is in eight, eight years. What I have had is people having late haematogenous infection maybe two or three, four years after a joint replacement. And that is something which is, which is unavoidable in that people get infections elsewhere and then they infect the joint. It's not really a surgeon's fault but I don't think any people should be getting early post-operative infection and that is a failure of er, the system somewhere. (Surgeon 4) I guess what you have to reassure yourself is there are so many other factors that can occur not only just you know, you and the operation, it’s just the whole process of sterilisation, disinfection, the patient factors, there are so many things that have problems, but you know, personally speaking …But at the same time, if a recurrent problem is happening then you need to step back and reflect and work out what’s going on. (Surgeon 2) I’m honest with my patients, you know, when they come to me with infections from elsewhere to say that every surgeon gets infections. I do it myself and I’m brutally honest with them about my er, rates personally for my clearance rates when I do infection work, and at the likelihood of recurrence in the long run. (Surgeon 3) It’s pretty devastating because you never really know why it happened, I mean fortunately, well touch wood, I haven’t had any primary joint replacements yet which have got infected, and I’ve done about 250 or so now, but I’m sure it will happen, you know, there’s no escaping it but there are so many. (Surgeon 2) It does, it knocks you for six, and you then start to look at practice again. You go back to the literature and you listen to things in meetings. Should I be doing this? Does it make a difference? You then start to look at things like skin prep; anything from that to what you’re closing the wound with. So, the whole process you re-examine again, not necessarily changing but just having your eyes open as to where the contamination is coming from. Is it me? Is it the pre-operative process? Have we missed something? (Surgeon 6) From a very selfish point of view it skew–erm, it will affect your figures on the registry. (Surgeon 10) We get appraised once a year and our infection rates are examined, in terms of locally, and our revision rates in terms of the NJR are looked at. (Surgeon 6)

**Table 2:** 

**Theme 2: A profound emotional impact**	It’s the kind of devastating…complication. Now with any infection you spend your whole life trying to avoid infection, and if the incidence of infection is, you know, whatever it is in your unit, so say it’s 0.5% of primary knee replacements and let’s say you do 100 per year, …you’re going to see one every two years, and for your entire career that might be–you might work for 30 years, you might have 15 infections. So, they don't happen very often, but you’ll probably remember every one. (Surgeon 11) Oh, it’s awful. It’s one of the worst things that can happen. [Really?] Beyond dying, yeah. No, it’s a catastrophe, absolute disaster. Awful, like you remember them by name… By-name, x-ray appearance and quite often organism as well. (Surgeon 5) It’s devastating…we pride ourselves in doing a good job and I think if you have one infection you can–I can virtually count them or remember their names in the last few years. (Surgeon 6) So a patient of mine gets a deep prosthetic joint infection, okay, well it’s happened once in nine years and that is horrible, really horrible you feel, er, you feel personally responsible. (Surgeon 8) On a personal level it’s horrible because you've harmed someone, well not necessarily you’ve harmed someone, but someone’s come to harm from something you’ve done. Erm, it’s obviously bad for the patient. It’s–it’s an unpleasant thing to have and they can have quite protracted treatment. (Surgeon 10) It’s devastating. Erm, you know, the er–my biggest paranoia is, is avoiding infection. (Surgeon 3) As a Surgeon, … who’s had complications, because we all do, you know, it’s pretty devastating you know, when something goes wrong, but you literally just have to get back on the horse and go in again otherwise it just eats you up and just destroys you, so you’ve just got to keep going. (Surgeon 2) It’s a bit soul-destroying really. You know, you do operations to try and make people better and you accept there are consequences that, you know, if you do enough operations then you will get complications, but it doesn't mean that it’s not, erm, a deeply unpleasant thing. (Surgeon 10)

**Table 3:** 

**Theme 3: Supporting each other**	I’m very fortunate to have, very excellent experts and, and nice colleagues really. So, you know, they–er, they don't so much come to me with problems cause I’m still very junior but, I’ve got several people that I can go to with problems and they’re very helpful and very supportive. So, it–it could be isolating but, it isn’t at [NHS Trust], for me anyway. (Surgeon 10) We have a weekly meeting where all the consultants come together, I chair it and, er, yeah, we just, it’s exactly the kind of thing we discuss…We talk about all the significant complications to be honest. (Surgeon 9) There’s a temptation to bury your head-in-the-sand because you don't want to accept that there’s a problem with something you’ve done. Erm, but, no, when you work in an environment where you’ve got, you know, essentially leaders in this area nationally, working, you know you’ve got support there if you get, you know, whatever problem you get, so that does make it easier. (Surgeon 10) The most important thing to do is to acknowledge it, not hide it under the carpet, be honest with your patient and because I work in a big unit I’ve got good colleagues in the knee group here and I work as part of an MDT with infection colleagues picking up the phone, talking to them [sure], getting advice, following a plan as part of the team. (Surgeon 8) I think it depends on where you’ve been, I’ve certainly worked in places where it would have been swept under the carpet and you know in a lot of places, most places orthopaedic surgeons are individuals and do practices and stuff that’s may be different to their colleagues and, and often you know don’t see colleagues necessarily, very frequently, they wouldn’t necessarily talk about these things so you're right a lot of this stuff wouldn’t even find out about. But that’s one of the reasons for having the meeting so that we can all talk about stuff and then we can all look at stuff and people can have, input from others, er, because sometimes you haven’t seen all the options and all the solutions or whatever and it’s useful to have input from other people and other people looking at stuff. (Surgeon 9)

### Theme 1: At some point infection is inevitable but surgeons still feel accountable

Participants described the nature of their work as one that they performed to alleviate the suffering of their patients. They also described how their work inherently involved risk and complications. Participants described dealing with a diagnosis of PJI as “devastating”, “soul-destroying” and “deeply unpleasant”, however they reported doing everything possible to minimise risk and to avoid its occurrence. This included analysing preoperative and intraoperative processes such as sterilisation and surgical wound closure. Despite careful inspection and planning, participants perceived PJI as an inevitability, suggesting that “despite every measure” it “happens to every surgeon” at some point in their career. One surgeon suggested that late, haematogenous PJI is unavoidable in patients with infections which migrate to the joint, whereas early post-operative PJI should be preventable. Another suggested that his feeling of devastation in response to a patient’s diagnosis of PJI was in part due to his lack of knowledge regarding the source of the patient’s infection. Surgeons felt strongly however, that adverse events were a consequence of their profession, and PJI an inevitable outcome at some point in every surgeons’ career when performing high numbers of joint replacement operations, and providing all appropriate measures are taken, they do not need to accept fault when it does occur. Surgeons appeared to expect a diagnosis of PJI at some point in their career, yet still its occurrence made them question their practice despite taking ‘every measure’ and left them feeling that they had let the patient down. This sense of accountability is also reinforced by the recording of individual surgeons’ clinical performance outcomes, which are scrutinised by the National Joint Registry, the hospital, their colleagues and the public, which left surgeons with a sense of ‘skewed’ accountability, despite the fact that PJI is rarely attributable to a failing of the surgeon.

Surgeons expressed the importance of being empathic and honest with their patients following a diagnosis of PJI and discussed their own need to engage in careful reflection on their practice, questioning their performance and re-examining all surgical processes. Participants suggested that the need to persevere and continue to perform surgery after a diagnosis of PJI was vital to ensuring they maintained their confidence.

### Theme 2: A profound emotional impact

Although the number of cases of PJI a surgeon may experience is relatively small in comparison to the number of operations they perform, the consequences of PJI are devastating for both patients and surgeons. Surgeons recognise the implications of PJI for patients and the impact that the infection and protracted treatment can have on their lives: “the worst thing to happen … beyond dying”, “a catastrophe, absolute disaster”. The consequences for patients are of such significance that surgeons were able to recall patients diagnosed with PJI, years after they had been treated, stating how they “remember every one”, “by name, x-ray appearance and quite often organism”. One surgeon described avoiding infection as his “biggest paranoia”. Participants discussed the pride they took in “doing a good job”. Another surgeon described how he could not help feeling that a patient with PJI had “come to harm” as a consequence of undergoing knee replacement, i.e. it would not have occurred if the joint had not been replaced. This suggests that surgeons feel some level of personal responsibility despite being unable to completely control for the occurrence of PJI. They were keenly aware of the consequences for patients and as such PJI was “devastating” and “deeply unpleasant” when it occurred. Two surgeons mentioned that such occurrences were “soul-destroying” and could “eat[s] you up and just destroy[s] you” unless they proved their resilience by maintaining their practice. This suggests that the occurrence of PJI has a significant emotional impact on surgeons, who feel responsible for patients’ well-being and above all pride themselves in doing their job well, to relieve patients of pain and improve their quality of life, and yet the occurrence of PJI could significantly worsen a patient’s quality of life. Surgeons stressed the importance of being resilient, maintaining their practice and accepting that such occurrences were inevitable at some point in their career.

### Theme 3: Professional consequences and need for support

When PJI does occur, surgeons highlighted the importance of acknowledging the diagnosis and being honest with patients. Surgeons have a high degree of professional autonomy and our findings suggest that in some departments decisions about treatment may be made individually and not always discussed with colleagues: one surgeon suggested that there was a temptation to “bury your head in the sand because you don't want to accept that there’s a problem with something you’ve done” but suggested he was lucky enough to work in a department with national leaders who specialised in PJI, and so felt supported. This may not always be the case for other surgeons. Another suggested that occurrence of PJI could potentially be “isolating” for a surgeon where such support did not exist.

All participants in this study reported having expert colleagues, who provided help and professional support. Surgeons reported that asking colleagues for advice and assistance and coming together as part of a strong and supportive team made dealing with a patient with PJI “easier”, and every participating surgeon described a need for and experience of being supported by colleagues, predominantly through regular, open discussions of surgical complications, within a multidisciplinary team. It is noteworthy however that one surgeon suggested that he had worked in departments where surgeons may not be supported as they might have wished.

## Discussion

Most patients improve after knee arthroplasty and do not experience any major adverse events, which makes it all the more difficult when something does go wrong. The surgeons in this study described how they are affected, emotionally and professionally when a patient they have treated develops a PJI, often without an obvious cause. Surgeons may experience ‘moral injury’ when their intention to help patients results in notable harm. While improvements in surgical techniques and health systems have greatly reduced the risk of PJI, surgeons still feel some level of personal responsibility despite being unable to completely control for its occurrence. Surgeons discussed how each case of PJI was memorable to them and described how important it is to come together as a team to discuss cases and support decisions around treatment. They acknowledged that this was not the case in all departments. Although surgeons worked with autonomy, they also relied on their team. Surgeons who experienced a strong and supportive team in which one could be open and honest with colleagues about a diagnosis of PJI, felt this enabled them to more effectively manage their surgical response to PJI.

Surgeons can feel vulnerable and isolated following an adverse event associated with surgeon error [9,10] or following an adverse event of multifactorial origin as shown in this study. The impact is shown to be greater when the complication is unexpected, results from elective surgery and leads to severe disability [14]. Such events can lead to intense emotional responses, impaired decision making, difficulty concentrating, a negative impact on clinical judgement and deleterious changes to practice [9,10]. The cumulative effect of these reactions may potentially impact upon surgeons’ personal and professional identities [10], and lead to a greater risk of error and suboptimal patient care [34]. Evidence suggests that surgeons may become increasingly conservative and risk-averse in response to these events [14], potentially reducing access to care for high-risk patients.

We suggest that uncertainty about the cause of PJI may greatly contribute to the impact on surgeons whilst increasing their vulnerability. The risk of PJI can be ameliorated, which explains why its occurrence is relatively uncommon, and this perhaps introduces a more profound level of disappointment when it does occur.

The findings of our study are important when considering the phenomenon of burnout syndrome [35]–a physical, emotional or mental exhaustion–that is common among surgeons [36–42]. One third of surgeons show high levels of burnout, regardless of their speciality [43]. Given the profound impact of PJI on surgeons, it is plausible to suggest that without adequate support, surgeons who treat PJI are at an increased risk of burnout and further adverse events [40].

Limited information exists about support interventions for health care professionals, in particular orthopaedic surgeons. Institutional support in the context of surgical complications has been described as inadequate, with a presence of strong blame cultures [14]. The surgeons in our study described the importance of accepting their role in the aetiology of infection, being honest with themselves and their patients, and reflecting on their practice. We suggest that the surgical response to PJI may be framed in a more positive light as a growth experience, and that a team and systems approach is crucial to ensure that surgeons support one another after adverse events. We also suggest this includes the wider surgical team. Providing teamwork and leadership training to clinicians, as well as educating clinicians about errors and adverse occurrence, are suggested as a means of primary preventative intervention to address clinicians’ increasing levels of stress, with secondary interventions including counselling and psychotherapy [44]. While the surgeons in our study did not express any concerns related to stress or burnout, the long-term impact on surgeons of a diagnosis of PJI suggests that these primary and secondary preventative interventions may be beneficial to orthopaedic surgeons. In January 2017, NHS England launched a health service for GPs with mental health concerns including stress and depression [45]. To our knowledge, there are no dedicated health services specifically developed to meet the needs of health care professionals. The Royal College of Surgeons provides “mentoring arrangements”, however, these are broad in remit and not specifically designed to support surgeons through adverse events or errors, although the college also provides a Confidential Support and Advice Service–a telephone helpline, where surgeons can discuss issues of concern [46].

Although we acknowledge the relatively small number of participants in this study, in keeping with qualitative methodology data saturation was achieved [47] and evidenced by the consistency with which all surgeons stressed the magnitude of the impact of knee PJI on themselves and their patients, speaking candidly about their experiences. Our team-approach in analysing the data ensured a rigorous analysis process [33]. On the basis of these two aspects of study design and conduct we are confident that the findings as presented here are an appropriate reflection of the dataset as a whole and will have relatively good transferability to other surgeons who experience PJI in patients whom they have treated [48]. Surgeons in this study were asked to describe how they are affected personally and professionally when their patients develop PJI. This was explored in the context of a wider interview exploring their experiences of treating knee PJI. It is possible that surgeons’ reflections were influenced by the passage of time, the support they may, or may not have received and the outcome of the patients they treated. We propose that Tables 1–3 provide a source of acknowledgement of the profoundly negative impact that occurrence of PJI has on surgeons, and that other surgeons may have shared similar experiences.

We believe that this is the first study to explore the impact of knee PJI on surgeons’ personal and professional well-being. Previous work has focused on major and minor adverse events, surgical complications, and medical error often across a variety of surgical disciplines [8–16]. While other studies have explored surgeons’ emotional reactions to a variety of adverse events and complications our study focussed solely on the occurrence of PJI. Similar to these other studies, our findings suggest that emotional reactions of orthopaedic surgeons to PJI are similar to surgeons’ reactions to a variety of other adverse events. PJI can be regarded as a major adverse event, which can be life-threatening. Previous studies have found that surgeons reactions to events are experienced more intensely when the outcome is major and the link of causality between surgeon contribution and event is more direct [10,14]. In most cases PJI cannot be attributed to surgeon error and this may mean that the personal impact on surgeons is less severe. In a qualitative interview study of vascular and general surgeons Pinto and colleagues found that inadequate institutional support and the existence of blame cultures also exacerbated the impact of complications on surgeons. While some surgeons in this study acknowledged that support may be lacking in some orthopaedic departments, based on their previous experiences, none suggested it was something they had experienced in their own current department. This may be because all of the participating surgeons in this study were from departments which specialised in treating PJI. Focusing solely on the personal and professional impact of PJI demonstrates the value of qualitative research for identifying areas for further research. We suggest that future studies should focus on identifying which aspects of PJI have the most impact on surgeons’ practice and personal wellbeing, and ways to further support surgeons to manage PJI.
